# Simulation-Based Assessment of the Impact of Non-Adherence on Endoxifen Target Attainment in Different Tamoxifen Dosing Strategies

**DOI:** 10.3390/ph14020115

**Published:** 2021-02-03

**Authors:** Anna Mueller-Schoell, Lena Klopp-Schulze, Robin Michelet, Madelé van Dyk, Thomas E. Mürdter, Matthias Schwab, Markus Joerger, Wilhelm Huisinga, Gerd Mikus, Charlotte Kloft

**Affiliations:** 1 Department of Clinical Pharmacy and Biochemistry, Institute of Pharmacy, Freie Universitaet Berlin, 14163 Berlin, Germany; anna.mueller-schoell@fu-berlin.de (A.M.-S.); lena.klopp-schulze@fu-berlin.de (L.K.-S.); robin.michelet@fu-berlin.de (R.M.); gerd.mikus@fu-berlin.de (G.M.); 2 Graduate Research Training Program PharMetrX, 12169 Berlin, Germany; 3 College of Medicine and Public Health, Flinders University, Adelaide, SA 5042, Australia; madele.vandyk@flinders.edu.au; 4 Dr. Margarete Fischer-Bosch Institute of Clinical Pharmacology, Stuttgart, and University Tübingen, 70376 Tübingen, Germany; Thomas.Muerdter@ikp-stuttgart.de; 5 Dr. Margarete Fischer-Bosch Institute of Clinical Pharmacology, 70376 Stuttgart, Germany; Matthias.Schwab@ikp-stuttgart.de; 6 German Cancer Consortium (DKTK), Partner Site Tübingen, German Cancer Research, 69120 Heidelberg, Germany; 7 Departments of Clinical Pharmacology, Pharmacy and Biochemistry, University Tübingen, 72076 Tübingen, Germany; 8 Department of Medical Oncology and Hematology, Cantonal Hospital, 9007 St. Gallen, Switzerland; Markus.Joerger@kssg.ch; 9 Institute of Mathematics, University of Potsdam, 14476 Potsdam, Germany; huisinga@uni-potsdam.de

**Keywords:** tamoxifen, non-adherence, model-informed precision dosing, pharmacokinetics, pharmacometrics

## Abstract

Tamoxifen is widely used in breast cancer treatment and minimum steady-state concentrations of its active metabolite endoxifen (C_SS,min ENDX_) above 5.97 ng/mL have been associated with favourable disease outcome. Yet, about 20% of patients do not reach target C_SS,min ENDX_ applying conventional tamoxifen dosing. Moreover, 4–75% of patients are non-adherent, resulting in worse disease outcomes. Assuming complete adherence, we previously showed model-informed precision dosing (MIPD) to be superior to conventional and *CYP2D6*-guided dosing in minimising the proportion of patients with subtarget C_SS,min ENDX_. Given the high non-adherence rate in long-term tamoxifen therapy, this study investigated the impact of non-adherence on C_SS,min ENDX_ target attainment in different dosing strategies. We show that MIPD allows to account for the expected level of non-adherence (here: up to 2 missed doses/week): increasing the MIPD target threshold from 5.97 ng/mL to 9 ng/mL (the lowest reported C_SS,min ENDX_ in *CYP2D6* normal metabolisers) as a safeguard resulted in the lowest interindividual variability and proportion of patients with subtarget C_SS,min ENDX_ even in non-adherent patients. This is a significant improvement to conventional and *CYP2D6*-guided dosing. Adding a fixed increment to the originally selected dose is not recommended, since it inflates interindividual variability.

## 1. Introduction

Tamoxifen is a selective modulator of the oestrogen receptor (ER), used for the treatment of ER-positive breast cancer in pre- and postmenopausal patients [1,2]. While it is also given in the neoadjuvant, palliative, and preventive setting, it is most often used for 5–10 years in the adjuvant setting [1,2]. The metabolism of tamoxifen is complex, involving several polymorphic enzymes such as CYP2D6, CYP3A5, CYP2C9, CYP2C19 as well as sulfotransferases and UDP-glucuronosyltransferases, respectively [3,4]. CYP2D6 plays a major role in the formation of endoxifen, tamoxifen’s most relevant and around 100-fold more active metabolite, resulting in a high interindividual variability in endoxifen minimum concentrations at steady-state (C_SS,min ENDX_) [5,6,7]. Tamoxifen is taken orally at 20 mg once daily (QD) and although convenient, the patient may behave non-adherently. Non-adherence, defined as <80% drug intake [8], is significantly associated with treatment failure [9,10,11]. A therapeutic target for C_SS,min ENDX_ of 5.97 ng/mL has been associated with superior survival [6,7]. However, at the conventional dose of 20 mg, patients with impaired or loss of CYP2D6 activity are at high risk of being below the target concentration, associated with a 26% higher breast cancer recurrence rate [7]. Reported adherence rates in adjuvant or preventive tamoxifen treatment vary from 25% to 96% [8,10,11,12,13,14,15,16] and associations made with non-adherence include young (<40–60 years) [12,15,17] and old age (>75–85 years) [10,12,15,18], married status [19], current or previous smoking [10,20,21], node-negative status [10], prior mastectomy [12], prior lumpectomy [15], comorbidities [15], prior history of thromboembolic events [10], sequential treatment assignment with aromatase inhibitors [10,14], adverse events [16,18,22,23,24] and a low socio-economic background [19,20].

In our previous work, we compared three different tamoxifen early dose finding regimens with an increasing degree of dose individualisation, assuming complete adherence (100% drug intake) [25]: Conventional dosing, *CYP2D6* genotype-predicted phenotype-guided dosing (short: *CYP2D6*-guided dosing) and model-informed precision dosing (MIPD) [26,27,28]. Amongst the three strategies, MIPD performed best both in terms of target attainment (92.8%, compared to 84.0% and 77.8% in *CYP2D6*-guided and conventional dosing, respectively) and lowest C_SS,min ENDX_ interindividual variability (IIV). Given the considerable concern of non-adherence to tamoxifen treatment, this study aimed to explore the impact of later non-adherence on C_SS,min ENDX_ target attainment in the three described and two additional modified MIPD early dose finding strategies using stochastic simulations. The simulation results showed that the risk for target non-attainment due to later non-adherence increased with increasing level of dose individualisation during the early dose finding stage. However, targeting the mean C_SS,min ENDX_ in *CYP2D6* genotype-predicted normal metabolisers (gNM) instead of the proposed therapeutic endoxifen threshold concentration, continued regular therapeutic drug monitoring, and including risk factors for non-adherence in existing pharmacokinetic/pharmacodynamic models used in MIPD can help preserve the value of MIPD in tamoxifen therapy, despite a high prevalence for non-adherence.

## 2. Results

**Figure 1:**
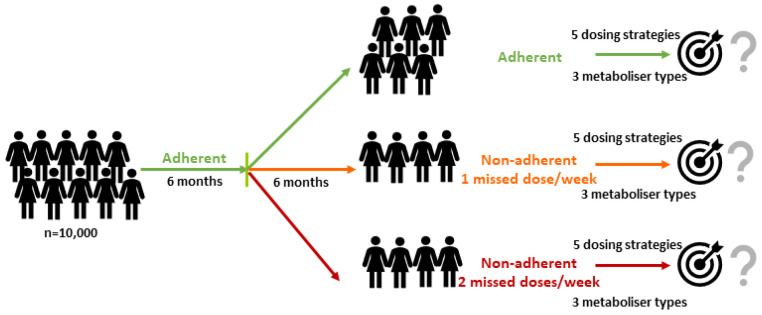
Workflow for the simulation study to assess the impact of two non-adherent scenarios compared to the full adherent scenario (0 missed doses/week, top) on endoxifen target attainment for five different dosing strategies comprising genotype-predicted normal metabolisers (gNM), intermediate metabolisers (gIM) and poor metabolisers (gPM).

Given the definition of non-adherence (<80% drug intake [8], translating into 1.4 missed tamoxifen doses per week), we simulated a large cohort of 10,000 virtual patients (in detail explained in the Methods section) amongst whom 60% were strictly adherent and 40% missed one or two consecutive tamoxifen doses per week over a period of six months following an initial six month period of complete adherence (Figure 1). The percentages of patients not reaching the proposed C_SS,min ENDX_ therapeutic threshold concentration of 5.97 ng/mL [7] in adherent and non-adherent patients were analysed for

(i) Conventional dosing (20 mg QD);
(ii) *CYP2D6*-guided dosing (gNM: 20 mg, gIM: 30 mg, gPM: 60 mg QD);
(iii) MIPD targeting the proposed 5.97 ng/mL (initial *CYP2D6*-guided dosing for 4 weeks, collection of virtual TDM samples at 2,3 and 4 weeks after treatment start and selection of maintenance dose after week 4 using Bayesian Forecasting);
(iv) MIPD targeting 5.97 ng/mL (dosing strategy (iii)) when adding 10 mg to each selected dose; and
(v) MIPD (dosing strategy (iii)) but targeting the lowest reported mean C_SS,min ENDX_ in gNM (9 ng/mL) [29].

The dosing strategy (iv) aimed to capture common practice upon observing subtarget concentrations or suspecting non-adherence. Conversely, dosing strategy (v) proposed a more individualised approach to account for later non-adherence in the MIPD early dose finding framework.

**Figure 2:**
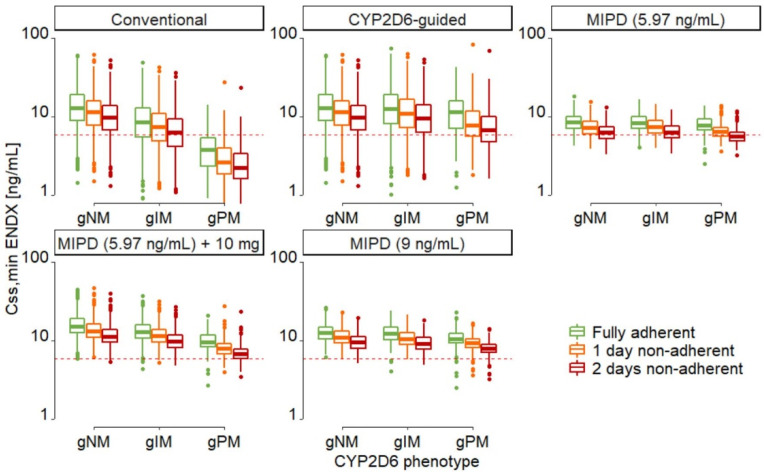
Minimum endoxifen concentrations at steady-state (C_SS,min ENDX_) for different *CYP2D6* genotype-predicted phenotypes in the five dosing strategies in strictly adherent patients (green), patients missing one dose per week (orange) and patients missing two consecutive doses per week (red) for six months, see Figure 1. Red dashed horizontal line: proposed endoxifen therapeutic threshold concentration (5.97 ng/mL) [7]; boxes: interquartile line (IQR) including median; whiskers: range from hinge to lowest/highest value within 1.5 IQR; points: data outside whiskers. Abbreviations: gNM, gIM, and gPM: genotype-predicted normal, intermediate, and poor metabolisers, respectively.

Individual dose selections (Supplementary Figures S1–S3), resulting in C_SS,min ENDX_, IIV and the risks for subtarget C_SS,min ENDX_ due to non-adherence were different amongst dosing strategies and across *CYP2D6* genotype-predicted phenotypes (Figure 2 and Supplementary Table S1).

In strictly adherent patients, the risks for subtarget C_SS,min ENDX_ were lowest in MIPD targeting C_SS,min ENDX_ of 9 ng/mL, and in MIPD targeting 5.97 ng/mL when adding 10 mg to each selected dose. The risk was moderately higher in MIPD targeting 5.97 ng/mL, followed by *CYP2D6* genotype-predicted phenotype-guided dosing and conventional dosing (Figure 2 green box-whisker plots, Table 1). The IIV was lowest in MIPD targeting C_SS,min ENDX_ of 5.97 ng/mL and 9 ng/mL, higher in MIPD targeting C_SS,min ENDX_ of 5.97 ng/mL when adding 10 mg to each selected dose, and highest in *CYP2D6*-guided and conventional dosing (Figure 2 and Supplementary Table S1).

**Figure 3:**
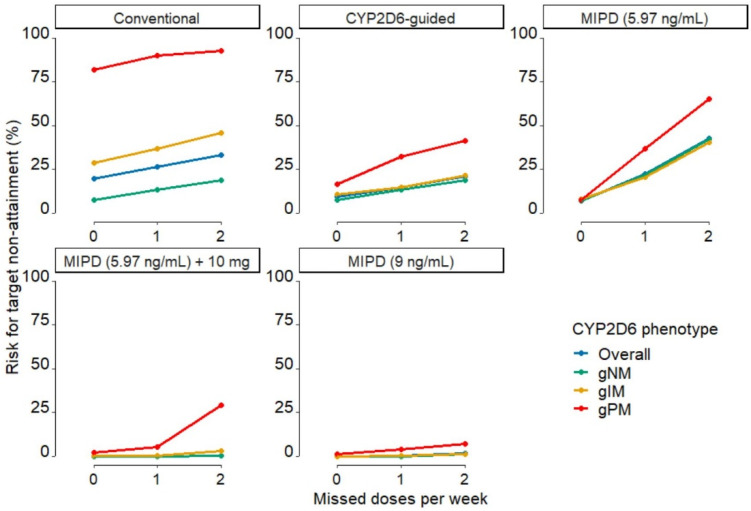
Risks for non-attainment of target minimum endoxifen concentrations at steady-state (C_SS,min ENDX_) in the different dosing regimens in fully adherent patients, patients missing one dose per week and patients missing two consecutive doses per week for six months. Green: gNM, yellow: gIM, red: gPM, blue: overall. Abbreviations: MIPD: model-informed precision dosing; gNM, gIM, and gPM: genotype-predicted normal, intermediate, and poor metabolisers, respectively.

When one or two consecutive doses per week were missed, relative risk increases, as assessed by the increase in risk relative to the baseline risk at complete adherence, were highest in MIPD approaches, moderate in *CYP2D6*-guided dosing, and lowest in conventional dosing (Table 2, Figure 3). The risks for target non-attainment in non-adherent patients were lowest in MIPD targeting 9 ng/mL and in MIPD targeting 5.97 ng/mL when adding 10 mg to each selected dose, while they were high in *CYP2D6*-guided and conventional dosing and highest in MIPD targeting 5.97 ng/mL.

Increases in risk for target non-attainment due to non-adherence increased with the increasing level of dose individualisation and were inversely proportional to the (absolute) risks for target non-attainment in strictly adherent patients. As expected, the risk of target non-attainment increased with the number of missed doses as well as with the impairment of CYP2D6 function (from gNM to gPM) (Table 1, Figure 3). Both modified MIPD dosing strategies resulted in lower percentages of gNM and gIM at risk. However, compared to MIPD targeting 9 ng/mL, MIPD targeting C_SS,min ENDX_ of 5.97 ng/mL when adding 10 mg to the selected dose resulted in a much higher IIV and a high percentage of non-adherent gPMs at risk. Conversely, MIPD targeting 9 ng/mL resulted in low risks in non-adherent patients across all genotype-predicted phenotypes. Furthermore, median C_SS,min ENDX_ were equivalent to the median C_SS,min ENDX_ simulated in *CYP2D6*-guided dosing but with significantly lower IIV (Supplementary Table S1). Thus, increasing the target level to 9 ng/mL presents a safeguard to the uncertainty associated with the patient status and physiology that is not captured and accounted for by covariates. Irrespective of the dose individualisation strategy chosen, strict adherence to tamoxifen intake is most critical in gPM patients.

## 3. Discussion

Non-adherence is often observed in patients undergoing long-term tamoxifen treatment and is a major concern due to its negative impact on disease outcome [9,10]. Furthermore, around 20% [7] of patients are considered at risk for C_SS_,_min ENDX_ below a proposed therapeutic threshold due to impaired CYP2D6 activity and additional high unexplained IIV. Of note, tamoxifen adherence could significantly increase the explained variability of endoxifen plasma concentrations in breast cancer patients [30]. An MIPD early dose finding framework has been proposed to increase the proportion of patients reaching target endoxifen concentrations and mitigate the high IIV observed in C_SS_,_min ENDX_ [25]. However, later non-adherence in long-term therapy is usually not considered in MIPD early dose finding frameworks. Continued regular TDM after initial dose titration could help to identify non-adherent patients early on. However, this service is also expensive, especially in long-term therapies, and is rarely feasible.

In this work, investigating the impact of non-adherence on different dosing strategies, we identified adherence as a crucial factor for the success of MIPD early dose finding strategies. While MIPD targeting the proposed therapeutic threshold concentration of 5.97 ng/mL is an excellent dosing strategy in strictly adherent patients, it performed worse than *CYP2D6*-guided dosing in patients missing one dose per week and was worst in patients missing two consecutive doses per week. In fact, almost half (42.8%) of MIPD patients targeting the therapeutic target threshold were at risk for subtarget C_SS_,_min ENDX_ when two consecutive doses were missed after the initial dose finding stage. This finding highlights an important feature of MIPD: precision dosing aims to identify the tailored dose for an individual patient to reach a target exposure. A common assumption is that every dose is taken correctly and, given that the dosing schedule is chosen to result in an exposure at but not significantly above the target and no uncertainty on adherence is taken into account, non-adherence will inevitably result in subtarget exposure. In our simulation study, conventional dosing showed the least relative sensitivity to non-adherence as assessed by the increase in risk relative to the baseline risk at complete adherence; however, the risks for target non-attainment were the highest in strictly adherent patients and patients missing one dose per week. Compared to conventional dosing, *CYP2D6*-guided dosing showed lower proportions of patients at subtarget C_SS_,_min ENDX_ in both non-adherence scenarios. Two factors can explain this: first, *CYP2D6*-guided dosing allows for the majority of patients to reach exposures above the target concentration. Second, because *CYP2D6*-guided dosing does not consider the IIV in patients within *CYP2D6* genotype-predicted phenotypes, large IIV in C_SS_,_min ENDX_ can be observed in *CYP2D6*-guided dosing (Figure 2, large range of whiskers). This results in many individual concentrations being significantly above the target concentration in adherent patients and lower, but not yet subtarget, concentrations in non-adherent patients. Yet, as some individuals experience very high C_SS_,_min ENDX_, which can lead to increased frequencies of adverse events [31,32,33], *CYP2D6*-guided dosing should not be the dosing strategy of choice.

To further strengthen the advantages of MIPD by implementing a safeguard to non-adherence, we explored two additional MIPD dosing strategies: dosing strategy (iv) adding a fixed increment (here: 10 mg) to each individual selected dose in MIPD using the original target, as it is commonly done in practice, or a dosing strategy (v) targeting a higher C_SS_,_min ENDX_ than the proposed therapeutic target threshold, i.e., the mean C_SS_,_min ENDX_ in gNM. There was a wide range (9.30–34.9 ng/mL) [29,34,35,36,37,38] of reported mean C_SS_,_min ENDX_ in gNM (formerly defined as genotype-predicted extensive metabolisers (gEM)) at conventional dosing from which, as the most conservative approach, we chose the lowest value (9 ng/mL [29]) for the increased PK target. In our simulations, both additional dosing strategies resulted in reduced risks due to non-adherence **(**Figure 3) and overall C_SS_,_min ENDX_ within the range of the C_SS_,_min ENDX_ observed in conventional dosing (Figure 2, Supplementary Table S1). Yet, due to the much higher IIV observed in dosing strategy (iv) (additional 10 mg), introduced by the fixed dose increment, and the large risk increase for gPM when missing two consecutive doses per week, this inferior approach cannot be recommended. To prevent the large increase in IIV, a relative rather than an absolute dose increase would be needed. Based on our simulation results, applying MIPD with a higher target C_SS_,_min ENDX_ of 9 ng/mL in patients with a high risk for non-adherence seems favourable. MIPD targeting a C_SS,min ENDX_ of 9 ng/mL results in median C_SS,min ENDX_ similar to the ones observed in *CYP2D6*-guided dosing (12.4 ng/mL vs. 12.9 ng/mL, Supplementary Table S1). Moreover, due to the reduced IIV (24.1% CV vs. 56.8% CV, Supplementary Table S1) it allows for almost all adherent patients to reach the proposed therapeutic target concentration of 5.97 ng/mL (99.9% vs. 90.8%, Supplementary Table S1) with minimal risk increases due to non-adherence (1.55% vs. 21.1% of patients at risk when missing two consecutive doses per week, Table 2). Of note, the span of tamoxifen doses in dosing strategy (v) is high, ranging from 5 mg QD to 120 mg QD. While we limited our maximum dose to the highest dose tested without additional toxicities [36], the safety of our proposed dosing framework has to be confirmed in a clinical trial before it can be recommended for use in clinical routine.

Additional measures such as continued regular therapeutic drug monitoring after initial therapeutic drug monitoring-based dose titration can aid in promptly identifying non-adherent patients [11] and allows to keep using the original target C_SS_,_min ENDX_ of 5.97 ng/mL. Finally, existing MIPD approaches for chronic/long-term treatments should be extended (where appropriate) to account for the likelihood of non-adherence and patient characteristics associated with it. Of note, the endoxifen PK target of 5.97 ng/mL was proposed in a study, which did not account for non-adherence and allowed sample collection up until four years after breast cancer diagnosis [7]. Thus, it cannot be excluded that the cohort analysed in this study contained non-adherent patients. Yet, a similar study in pre-menopausal patients [6], in which non-compliant patients (7% of all patients) were excluded for the clinical endpoint analysis, identified a PK target very similar to the target proposed by Madlensky et al. [7] (5.29 ng/mL vs. 5.97 ng/mL) Thus, the possible bias due to non-adherent patients in the Madlensky study would be small. Nevertheless, a prospective well-designed trial with careful monitoring of adherence could aid in defining a PK target with no potential bias due to non-adherence. Independent from this, as this study focused on the impact of non-adherence on attaining a certain PK target instead of the exact numerical value of the PK target itself, a change in the PK target would not result in a change to our general findings.

Lastly, based on our pharmacokinetic model, our study was limited to the investigation of the impact of non-adherence on the tamoxifen/endoxifen exposure. Thus, given steady-state attainment under non-adherence, the total duration of non-adherence would not change the results of our study. However, as the total duration of non-adherence certainly impacts the overall risk for breast cancer recurrence, future studies using a pharmacodynamic model should focus on the impact of non-adherence and its duration on clinical endpoints.

## 4. Materials and Methods

A previously published joint parent-metabolite nonlinear mixed-effects pharmacokinetic (PK) model of tamoxifen and endoxifen [39] with its final parameter estimates was used for all simulations in this work. In short, the model consisted of a gut compartment from which tamoxifen was characterised to be absorbed in a first-order process (k_a_) with a lag time (t_lag_). Once absorbed, tamoxifen was characterised to distribute within a central compartment (V_TAM_/F) and to be either eliminated by linear formation of endoxifen (CL_23_/F) or by another linear elimination process (CL_20_/F) comprising other metabolic pathways than to endoxifen. The metabolite endoxifen was characterised to distribute in a central compartment (V_ENDX_/F) and to be eliminated in a linear process (CL_30_/F). Three covariate–PK parameter relationships were identified: the *CYP2D6* genotype, implemented as a fractional change model, had a significant impact on endoxifen formation (CL_23_/F), while patient age and body weight, both implemented as power models, significantly influenced the tamoxifen clearance to metabolites other than endoxifen (CL_20_/F). Interindividual variability components were implemented on the endoxifen formation and the tamoxifen clearance to other metabolites. Model development and the criteria used for it as well as an extensive covariate analysis, have been explained in detail in [25] and [39], respectively. The simulations were performed in NONMEM 7.4., called through Perl speaks NONMEM (PsN) v. 3.6.2 using the workbench Pirana v. 2.9.7 [40]. Pre- and postprocessing was performed in R v. 3.5.1, accessed through RStudio Version 1.2.1184, using packages *Xpose4, ggplot2, plyr, dplyr* and *zoo*.

To perform the simulation analyses, a large number of virtual breast cancer patients (*n* = 10,000), representing the same frequency of covariates (*CYP2D6* genotype, age, body weight) as observed in the clinical PK database (*n* = 1388 patients) used for model development, was generated. Concretely, representing the distribution of *CYP2D6* activity scores (AS) [41,42] in the model development dataset [39], the virtual population consisted of 56.6% *CYP2D6* genotype-predicted normal metabolisers (gNM), defined as AS ≥1.5 and including patients with missing AS imputed to AS 2, 37.8% genotype-predicted intermediate metabolisers (gIM), defined as AS 0.5-1 and 5.6% genotype-predicted poor metabolisers (gPM), defined as AS 0 [43]. Furthermore, for every virtual patient, a random age and body weight value was sampled with replacement from the age and body weight values recorded in the model development dataset.

The impact of one missed dose or two consecutive missed doses per week on endoxifen target (C_SS,min ENDX_ > 5.97 ng/mL [7]) attainment was compared for different dosing strategies with different levels of dose individualisation. Slightly modified from a previous investigation [25], the first three dosing strategies were: (i) conventional dosing (20 mg tamoxifen once daily (QD), (ii) *CYP2D6*-guided dosing (gNM: 20 mg QD, gIM: 30 mg QD (adjusted from 40 mg QD upon classification of AS 1 as gIM instead of gNM [43]), PM: 60 mg QD) and (iii) model-informed precision dosing (MIPD). The rationales for dosing strategies (i)–(iii) and detailed information on how MIPD was simulated were described before [25]. In MIPD, the initial dose was based on the *CYP2D6* genotype-predicted phenotype and the maintenance dose was selected using Bayesian Forecasting based on individual patient characteristics and three TDM samples taken at 2, 3 and 4 weeks after treatment start. This MIPD design was the result of previous systematic investigations regarding the optimal frequency and time points of TDM sampling [25]. Two additional dosing strategies were explored regarding their potential to increase the forgiveness of MIPD to non-adherence: (iv) MIPD targeting the proposed C_SS,min ENDX_ therapeutic threshold concentration of 5.97 ng/mL while adding a fixed increment (here: 10 mg) to each individual dose, as it is common in current clinical practice [33,37,38,44] and (v) MIPD targeting the lowest reported mean endoxifen C_SS_,_min ENDX_ in gNM (9 ng/mL) [29]. As it is common procedure to increase the dose in fixed increments, i.e., in 10 mg steps due to available tablet strengths, the dosing strategy (iv) aimed to represent the status-quo of dose adjustments to increase drug concentrations in clinical practice [33,37,38,44]. Representing the common clinical practice to measure minimum concentrations [45], we chose to collect our virtual TDM samples at the end of a dosing interval. However, as fluctuations in endoxifen concentrations within a steady-state dosing interval are minimal [46], our results are also applicable to different times of sample collection once a steady-state has been attained.

To assess the impact of late non-adherence on endoxifen target attainment, 6 months of full adherence (100% drug intake) were simulated for all dosing strategies (*n* = 10,000 patients each), assuring endoxifen steady-state attainment in all patients. Next, based on a previous report [19] and to include a sufficient number of patients in both groups, 60% of patients were simulated to stay fully adherent for the following 6 months. For the remaining 40%, two scenarios were simulated, in which patients missed either one dose or two consecutive doses per week (corresponding to the non-adherence definition of <80% correct medication intake [8] or 1.4 missed doses per week). Of note, in the second 6 months, patients continued to receive the dose determined at “treatment start”, i.e., in the first 6 months of complete adherence. No additional TDM samples were taken during the second 6 months. After a total of 12 months, assuring new steady-state attainment under non-adherence, the proportion of patients with C_SS,min ENDX_ below the target 5.97 ng/mL (i.e., the number of patients at risk) in the adherent and non-adherent groups in all dosing strategies were assessed overall and for different *CYP2D6* genotype-predicted phenotypes specifically.

## Figures and Tables

**Table 1:** Percentage of strictly adherent patients at risk (%) for target non-attainment.

Patient Subpopulation	Conventional Dosing	*CYP2D6*-Guided Dosing	MIPD (5.97 ng/mL Target)	MIPD (5.97 ng/mL Target) +10 mg	MIPD (9 ng/mL Target)
Overall ^†^	19.8	9.19	7.34	0.233	0.133
gNM	7.60	7.60	6.98	0.0294	0.00
gIM	28.9	10.5	7.85	0.220	0.132
gPM	81.7	16.5	7.51	2.40	1.50

Abbreviations: g*X*M: genotype-predicted metaboliser; MIPD: model-informed precision dosing, NM: normal metaboliser, IM: intermediate metabolisers; PM: poor metaboliser; ^†^: For prevalence of different genotype-predicted phenotypes, see Methods section; bold: dosing strategy with lowest percentage of patients at risk.

**Table 2:** Number of patients at risk (%) for target non-attainment due to missing doses.

	Conventional Dosing		*CYP2D6*-Guided Dosing		MIPD (5.97 ng/mL Target)		MIPD (5.97 ng/mL Target) +10 mg		MIPD (9 ng/mL Target)	
Number of missed doses	1	2	1	2	1	2	1	2	1	2
Overall	26.4	33.3	14.8	21.1	22.3	42.8	0.525	3.02	**0.375**	**1.55**
gNM	13.2	19.0	13.2	19.0	22.1	42.1	**0.00**	**0.530**	0.132	1.15
gIM	36.8	45.8	14.8	21.3	20.5	40.4	0.594	2.91	**0.198**	**1.32**
gPM	90.1	92.8	32.4	41.4	36.9	65.3	5.41	29.3	**4.05**	**7.21**

Abbreviations: gNM, gIM, and gPM: genotype-predicted normal, intermediate, and poor metabolisers, respectively. Bold: dosing strategies with lowest percentage of patients at risk having missed one or two doses, respectively.

## Data Availability

The simulation datasets presented in the current study are available from the corresponding author upon reasonable request.

## Supplementary Materials

The following are available online at https://www.mdpi.com/1424-8247/14/2/115/s1, Figure S1: Range and proportions of the individual dose selection in MIPD targeting the proposed therapeutic target threshold of 5.97 ng/mL, Figure S2: Range and proportions of the individual dose selection in MIPD targeting the lowest reported mean C_SS,min ENDX_ in gNM of 9 ng/mL, Figure S3: Range and proportions of the individual dose selection in MIPD targeting the proposed therapeutic target threshold of 5.97 ng/mL and adding 10 mg to each selected dose, Table S1: Comparison of C_SS,min ENDX_, interindividual variability and proportion in fully adherent patients with subtarget concentrations in the five dosing strategies stratified in the three CYP2D6 genotype-predicted phenotypes with their individual doses.
