# Supplementary material for: Simulation-Based Assessment of the Impact of Non-Adherence on Endoxifen Target Attainment in Different Tamoxifen Dosing Strategies
Source: Pharmaceuticals (Basel). 2021 Feb 3;14(2):115. doi: 10.3390/ph14020115 (PMC7913149; doi:10.3390/ph14020115)
Supplement: Supplementary file 1 [file pharmaceuticals-14-00115-s001.pdf]

Supplementary material

Simulation-based Assessment of the Impact of Non-adherence on Endoxifen Target Attainment in Different Tamoxifen Dosing Strategies

Authors: Anna Mueller-Schoell, Lena Klopp-Schulze, Robin Michelet, Madelé van Dyk, Thomas E. Mürdter, Matthias Schwab, Markus Joerger, Wilhelm Huisinga, Gerd Mikus and Charlotte Kloft

Table S1. Comparison of $C_{SS,min\ ENDX}$, interindividual variability and proportion in fully adherent patients with subtarget concentrations in the five dosing strategies stratified in the three CYP2D6 genotype-predicted phenotypes with their individual doses.

	(1) Standard dosing				(2) CYP2D6-guided dosing				(3) Model-informed precision dosing (5.97 ng/mL target)				(4) Model-informed precision dosing (5.97 ng/mL target) + 10 mg				(5) Model-informed precision dosing (9 ng/mL target)			
**CYP2D6**	gNM	gIM	gPM	all	gNM	gIM	gPM	all	gNM	gIM	gPM	all	gNM	gIM	gPM	all	gNM	gIM	gPM	all
$n_{patients}$	3396	2269	333	5998	3396	2269	333	5998	3396	2269	333	5998	3396	2269	333	5998	3396	2269	333	5998
**Dose (mg)**	20	20	20	20	20	30	60	20-60	5-120	5-120	5-120	5-120	15-130	15-130	15-130	15-130	5-120	5-120	5-120	5-120
$C_{SS,minENDX}$																				
**-Median**	13.1	8.52	3.81	10.7	13.1	12.8	11.4	12.9	8.52	8.39	7.74	8.41	15.3	13.0	9.71	14.1	12.7	12.3	10.6	12.4
**-IQR**	9.06-19.1	5.48-12.8	2.36-5.36	6.80-16.2	9.06-19.1	8.22-19.2	7.07-16.1	8.65-19.0	7.05-10.2	7.06-9.98	6.80-9.29	7.03-10.1	12.8-19.0	10.8-15.8	8.33-11.9	11.5-17.6	10.6-15.0	10.4-14.8	9.48-12.4	10.4-14.8
**-CV, %**	54.0	60.5	57.2	62.5	54.0	60.5	57.2	56.8	24.2	24.2	22.6	24.2	30.1	29.4	25.8	32.0	23.7	24.0	22.7	24.1
$n_{risk,\%}$	7.69	28.9	81.7	19.8	7.60	10.5	16.5	9.19	7.60	10.5	16.5	9.19	0.0294	0.220	2.40	0.233	0.00	0.132	1.50	0.133

Abbreviations: $C_{SS,min\ ENDX}$: minimum endoxifen concentrations at steady-state [ng/mL]; CV: coefficient of variation; IQR: inter-quartile range; gXM: genotype predicted metaboliser; NM: normal metaboliser; IM: intermediate metabolisers, PM: poor metabolisers; n_{risk}, %: proportion of patients at risk for $C_{SS,min\ ENDX}$ below the proposed therapeutic threshold of 5.97 ng/mL [1]

Figure S1. Range and proportions of the individual dose selection in MIPD targeting the proposed therapeutic target threshold of 5.97 ng/mL [1].

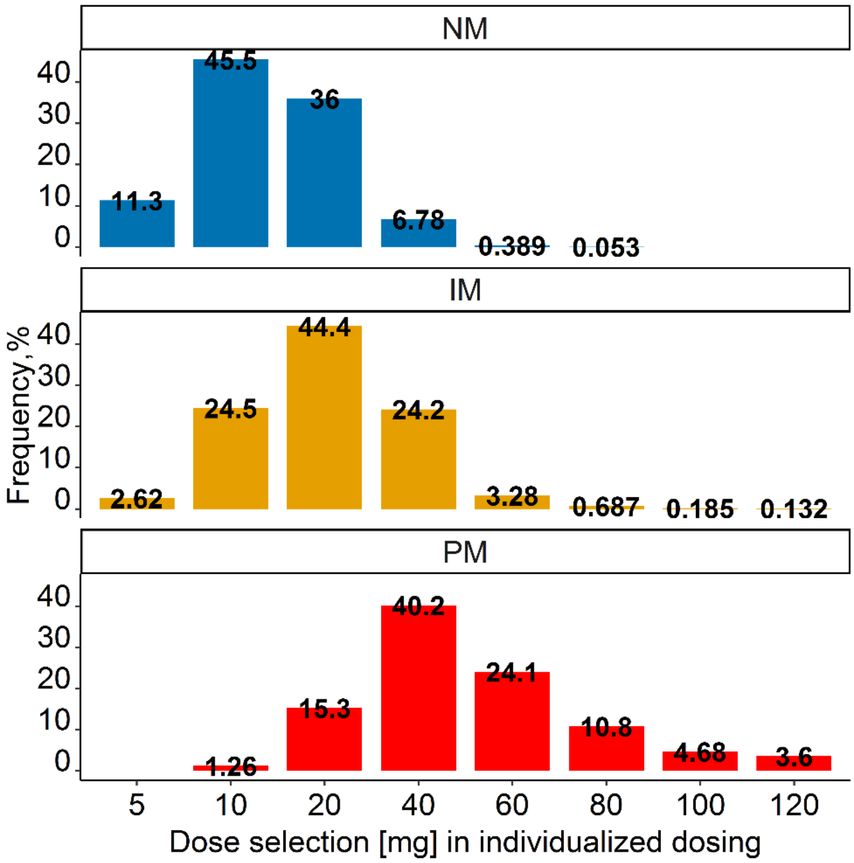

Abbreviations: gXM: genotype predicted metaboliser; NM: normal metaboliser; IM: intermediate metabolisers, PM: poor metabolisers.

Figure S2. Range and proportions of the individual dose selection in MIPD targeting the lowest reported mean $C_{SS,min\ ENDX}$ in gNM of 9 ng/mL [2].

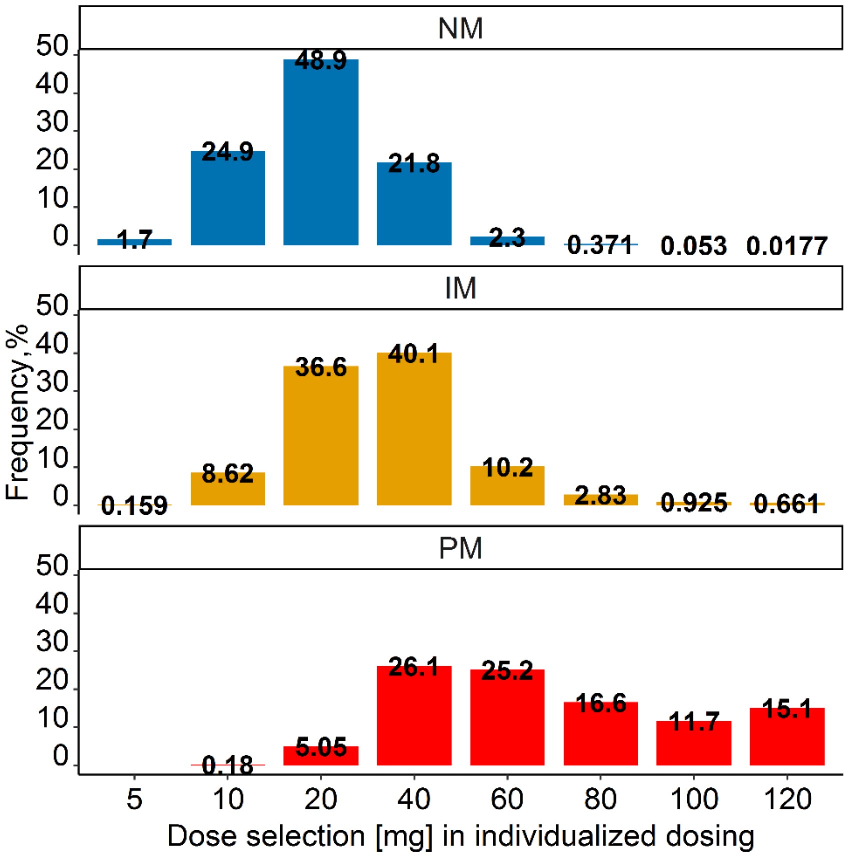

Abbreviations: gXM: genotype predicted metaboliser; NM: normal metaboliser; IM: intermediate metabolisers, PM: poor metabolisers.

Supplementary Figure 3. Range and proportions of the individual dose selection in MIPD targeting the proposed therapeutic target threshold of 5.97 ng/mL [1] and adding 10 mg to each selected dose.

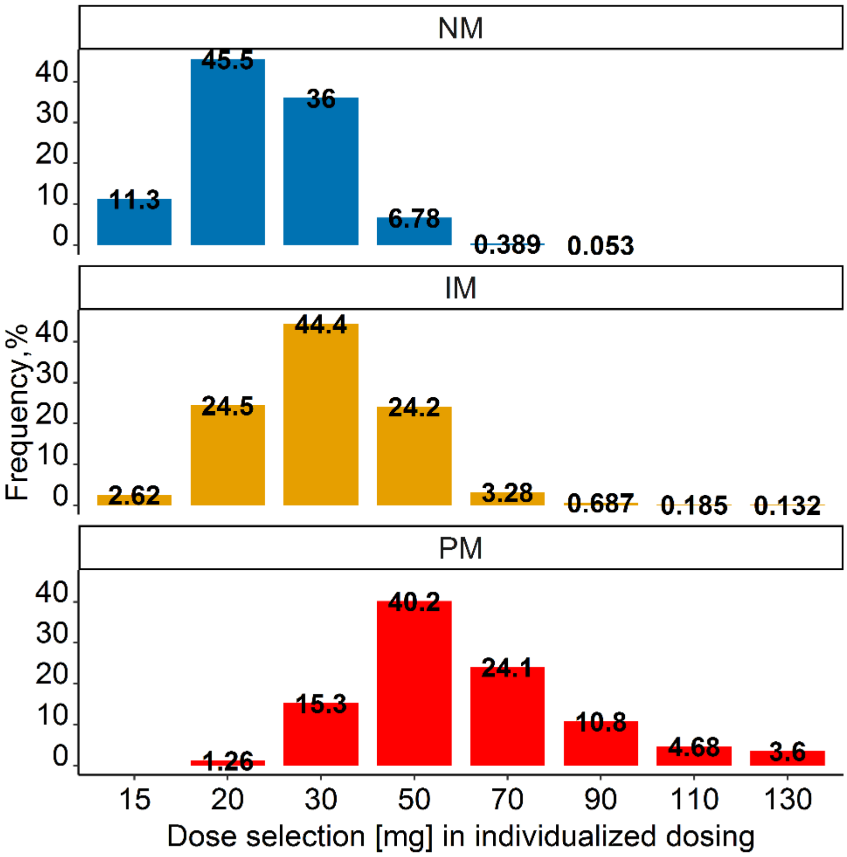

Abbreviations: gXM: genotype predicted metaboliser; NM: normal metaboliser; IM: intermediate metabolisers, PM: poor metabolisers.

References

1. Madlensky, L.; Natarajan, L.; Tchu, S.; Pu, M.; Mortimer, J.; Flatt, S.W.; Nikoloff, D.M.; Hillman, G.; Fontecha, M.R.; Lawrence, H.J.; et al. Tamoxifen metabolite concentrations, CYP2D6 genotype, and breast cancer outcomes. *Clin. Pharmacol. Ther.* **2011**, *89*, 718–725, doi:10.1038/clpt.2011.32.

2. Hertz, D.L.; Deal, A.; Ibrahim, J.G.; Walko, C.M.; Weck, K.E.; Anderson, S.; Magrinat, G.; Olajide, O.; Moore, S.; Raab, R.; et al. Tamoxifen Dose Escalation in Patients With Diminished CYP2D6 Activity Normalizes Endoxifen Concentrations Without Increasing Toxicity. *Oncologist* **2016**, *21*, 795–803.
